# Supplementary material for: The Spike Protein of SARS-coV2 19B (S) Clade Mirrors Critical Features of Viral Adaptation and Coevolution
Source: Microorganisms. 2022 Oct 12;10(10):2017. doi: 10.3390/microorganisms10102017 (PMC9609303; doi:10.3390/microorganisms10102017)
Supplement: Supplementary file 1 [file microorganisms-10-02017-s001.zip › microorganisms-1945647-supplementary.pdf]

microorganisms

Appendix A

Supplementary Tables and Figures:

Supplementary Table 1. The distribution in the 19B (S) lineages in the different contents, in which the A.23 and A.27 were dominating Africa with approximate percentages of 39.67% and 14.996% respectively, while the A Lineage dominated Asia with 61.1%. The A.2 found to be common in Europe, North America, South America and Oceania with approximate percentages of 37.23%, 40.623%, 76.23% and 56.93% respectively.

Countries / Lineages	Africa 1147	Asia 1349	Europe 3546	North America 8313	South America 244	Oceania 938
**A**	16.8%	61.1%	9.5%	9.1%	2.05%	10.7%
**A.1**	0	1.3%	1.2%	35.6%	4.5%	30.2%
**A.2**	0.35%	3.2%	37.2%	40.6%	76.2%	56.9%
**A.3**	0.09%	0.6%	0.03%	5.3%	0	0.32%
**A.4**	0	0.07%	0.03%	0.23%	0	0
**A.5**	0	0.6%	11.3%	0.21%	17.2%	0.53%
**A.6**	0	14.1%	0	0	0	0
**A.7**	0	0.82%	0	0	0	0
**A.9**	0	1.5%	0.03%	0	0	0
**A.11**	1.4%	0	0.03%	0	0	0
**A.12**	0.26%	0	0.03%	0	0	0
**A.15**	0	0	1.7%	0	0	0
**A.16**	0	0	0	0	0	0
**A.17**	0	0.07%	0.3%	0.02%	0	0
**A.18**	1.8%	0	0.03%	0.1%	0	0

**A.19**	8.1%	0.07%	0.17%	0.02%	0	0
**A.21**	9.07%	0.82%	4.5%	0.43%	0	0.32%
**A.22**	0.17%	0.82%	0	0	0	0
**A.23**	39.6%	5.8%	7.08%	7%	0	0.11%
**A.24**	0	0.96%	0	0	0	0
**A.25**	2.9%	0.07%	0.3%	0.01%	0	0.32%
**A.26**	0	0	0.37%	0	0	0
**A.27**	14.9%	0.3%	13.6%	0.1%	0	0.11%
**A.28**	1.7%	2.08%	8.8%	0.1%	0	0
**A.29**	2.2%	0.96%	3.6%	0.2%	0	0.43%
**A.30**	0.26%	0	0.06%	0	0	0

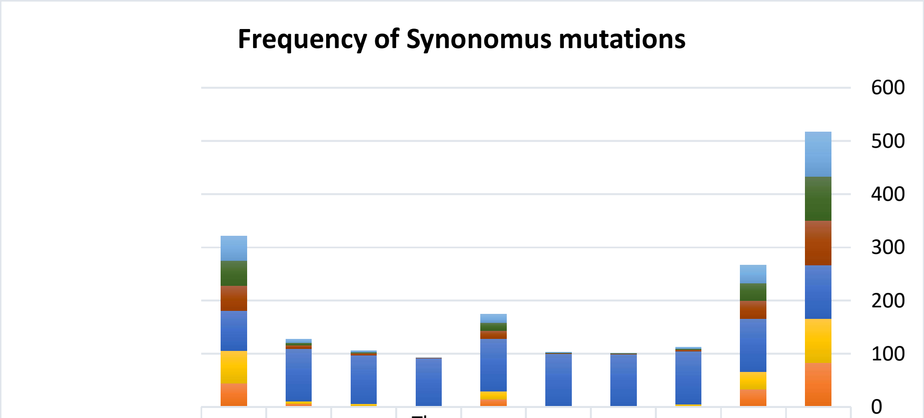

	Sudan	India	Germany	The United States of America	The United Kingdom	Canada	Australia	Belgium	Jordan	Gambia
NSP16:A199A	46.8	7.2	2.7	0.5	15.6	0.96	0.8	2.4	33.3	83.3
NSP16:K137K	46.8	5.07	2.7	0.3	14.7	0.84	0.64	2.4	33.3	83.3
NSP9:N95N	46.8	5.7	2.7	0.4	15.5	1.1	0.64	2.4	33.3	83.3
NSP4:S76S	74.8	98.5	92.5	90.9	98.7	98.4	98.3	100	100	100
NSP4:F17F	61.7	5.7	2.7	0.3	14.9	0.84	0.64	2.4	33.3	83.3
NSP2:N435N	44.6	5.7	2.7	0.5	14.7	0.96	0.64	2.4	33.3	83.3

Supplementary Figure 1. The frequency of the shared synonymous mutations in samples of 19B (S) clade A.29 lineage with different pattern among countries over the whole genome include NSP16:A199A, NSP16:K137K, NSP9:N95N, NSP4:S76S, NSP4:F17F, and NSP2:N435N.

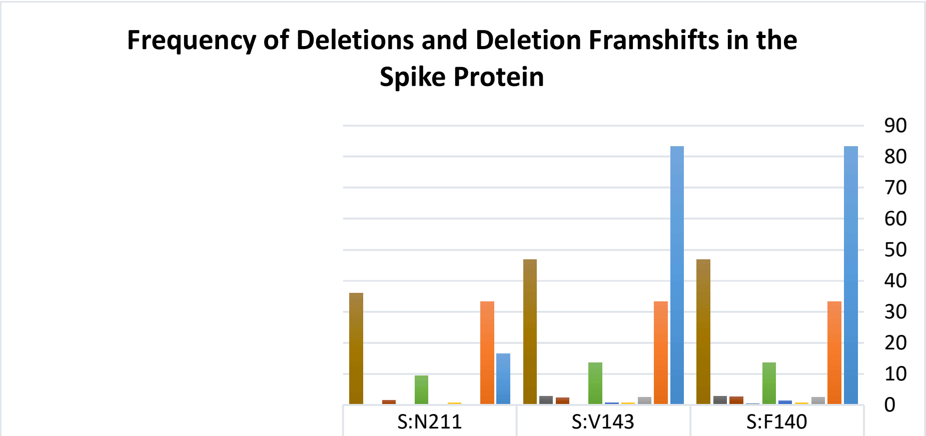

	S:N211	S:V143	S:F140
Gambia	16.6	83.3	83.3
Jordan	33.3	33.3	33.3
Belgium	0	2.4	2.4
Australia	0.64	0.64	0.64
Canada	0.18	0.84	1.4
The United Kingdom	9.5	13.6	13.6
The United States of America	0.2	0.3	0.4
Germany	1.5	2.3	2.7
India	0	2.8	2.8
Sudan	36.1	46.8	46.8

Supplementary Figure 2. The frequency of the shared Deletion and Deletion-Frameshifts in samples of 19B (S) clade A.29 lineage with different pattern among countries over the whole genome include S: F140, S: V143, S: N211.

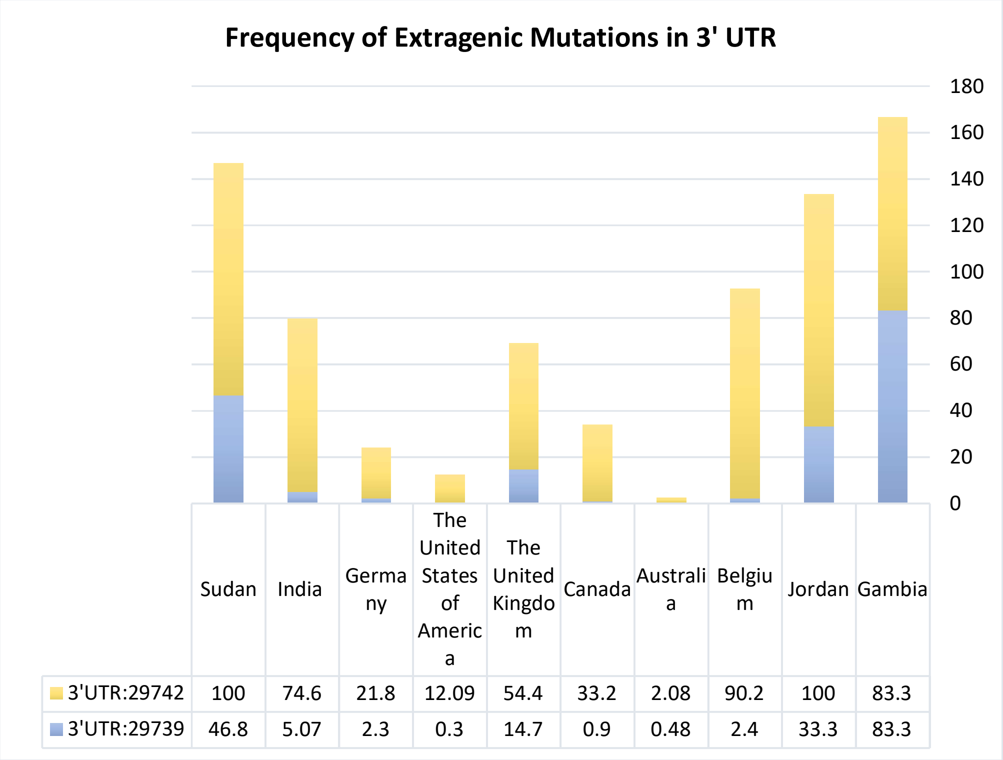

Supplementary Figure 3. The frequency of shared Extragenic mutations in the 3'UTR region in samples of 19B (S) clade A.29 lineage with different pattern among countries over the whole genome.

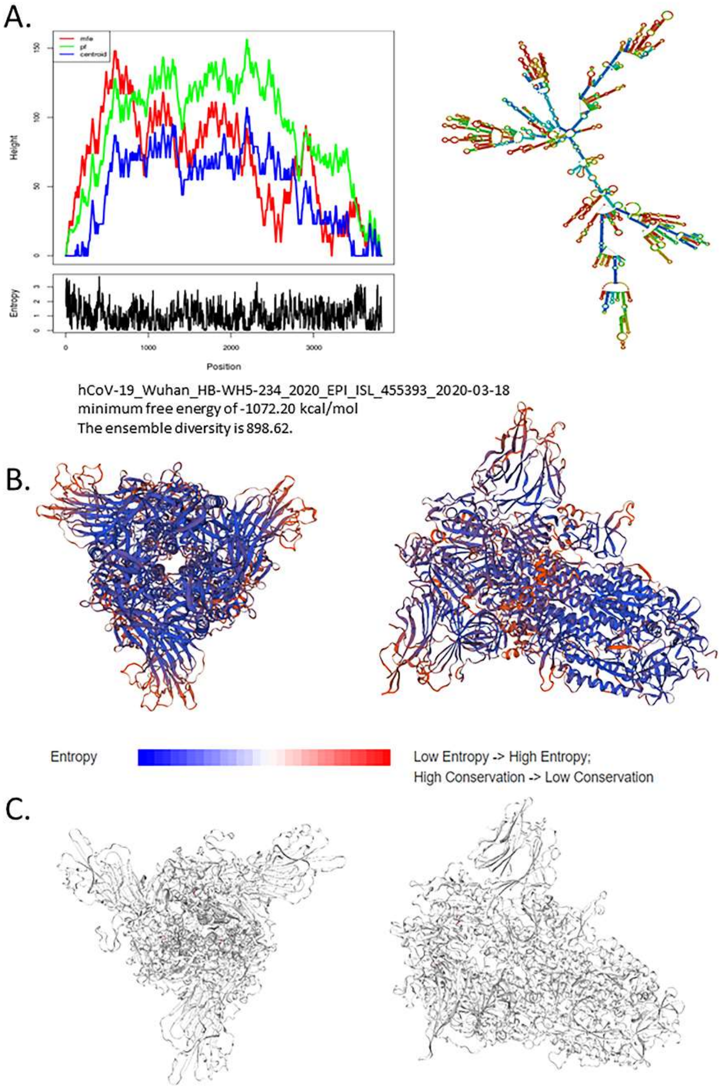

Supplementary Figure 4. A. Secondary structure, for the sample hCoV-19_Wuhan_HB-WH5-234_2020_EPI_ISL_455393_2020-03-18 (Reference: accession number NC 045512.2S), Minimum free energy of -1072.20 kcal/mol, the ensemble diversity is 898.62. B. 3D structure of the spike protein using the template 7cn8.1.A, seq identity: 91.68% with the description of Glycoprotein Cryo-EM structure of PCoV_GX spike glycoprotein. C. show no deletion in the spike protein.

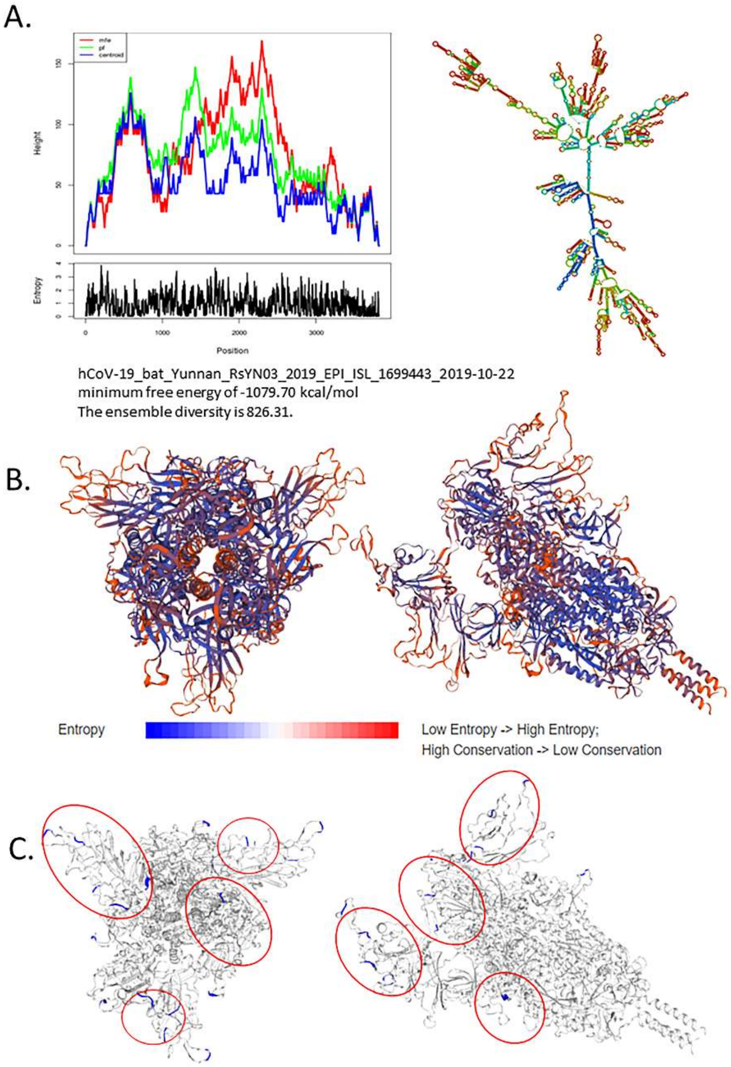

Supplementary Figure 5. A.Secondary structure for the sample hCoV-19_bat_Yunnan_RsYN03_2019_EPI_ISL_1699443_2019-10-22, Minimum free energy of -1079.70 kcal/mol, and the ensemble diversity is 826.31. B. 3D structure of the spike protein using the template 7sbo.1.A, seq identity: 78.48% with the description of Glycoprotein 1 RBD-up 2 of pre-fusion SARS-CoV-2 Delta variant spike protein. C. shows multiple deletions in the spike protein marked in blue.

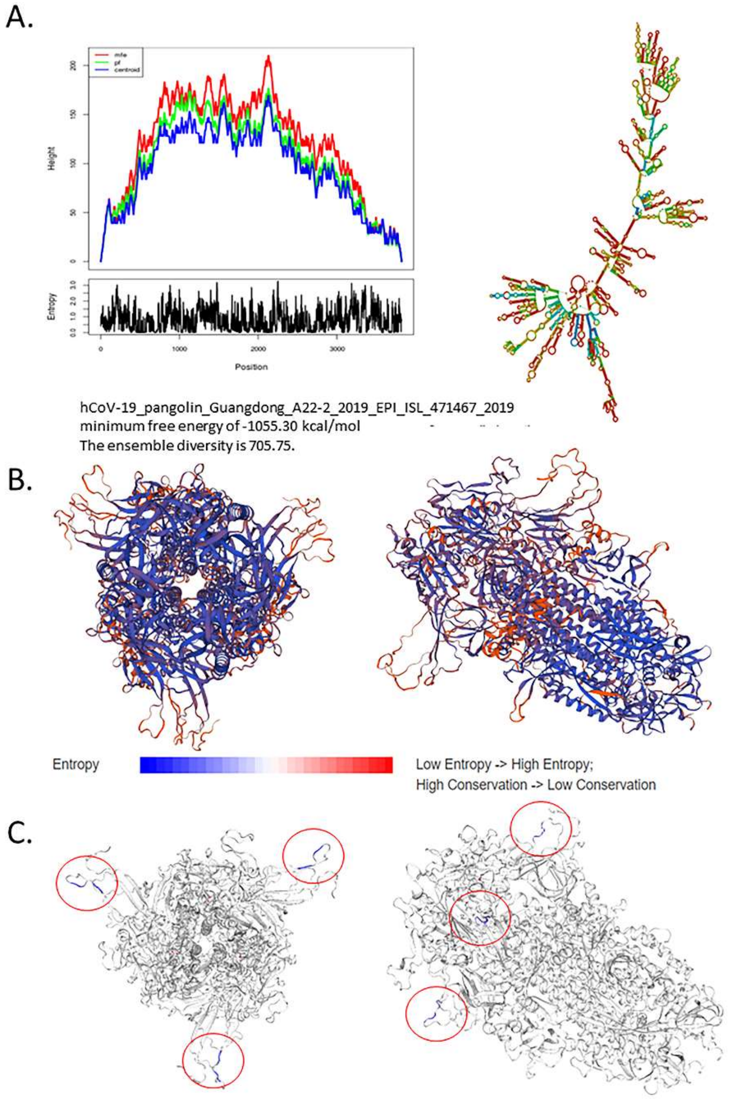

Supplementary Figure 6. A.Secondary structure for the sample hCoV-19_pangolin_Guangdong_A22-2_2019_EPI_ISL_471467_2019, Minimum free energy of -1055.30 kcal/mol, and the ensemble diversity is 705.75. B. 3D structure of the spike protein using the template 7cn8.1.A, seq identity: 90.73% with the description of Glycoprotein Cryo-EM structure of PCoV_GX spike glycoprotein. C. shows multiple deletions in the spike protein marked in blue.

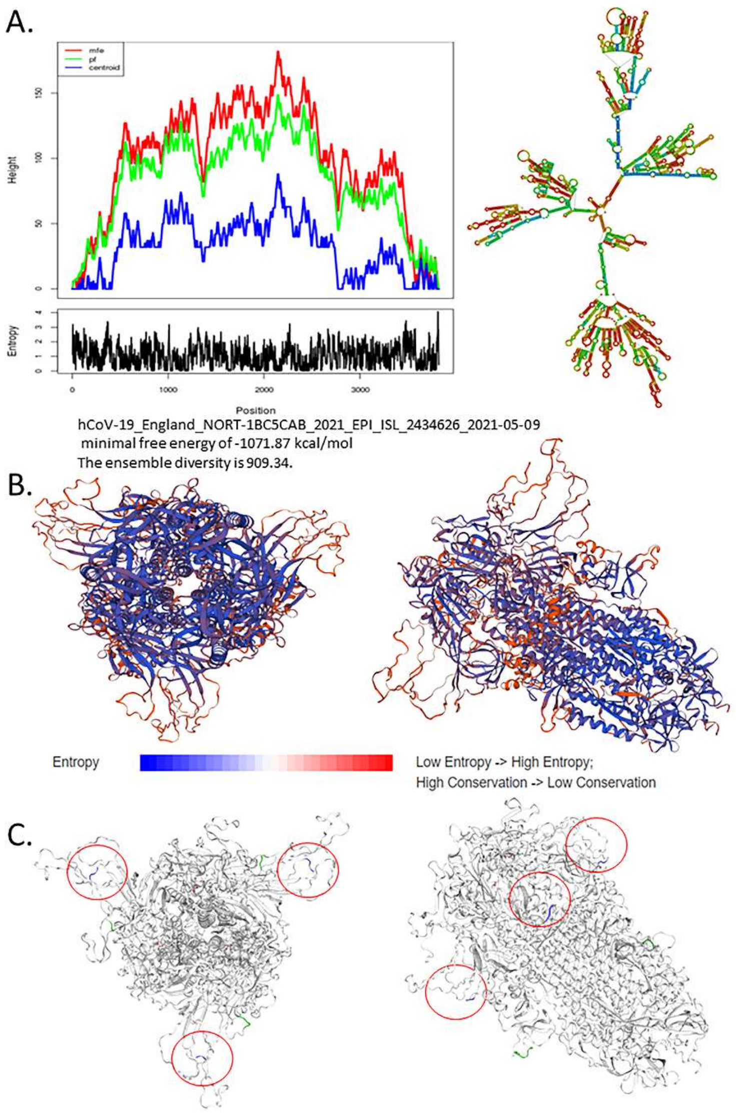

Supplementary Figure 7. A.Secondary structure for the sample hCoV-19_England_NORT-1BC5CAB_2021_EPI_ISL_2434626_2021-05-09, Minimal free energy of -1071.87 kcal/mol, and the ensemble diversity is 909.34. B. 3D structure of the spike protein using the template 7cn8.1.A, seq identity: 92.18% with the description of Glycoprotein Cryo-EM structure of PCoV_GX spike glycoprotein. C. shows multiple deletions in the spike protein marked in blue.

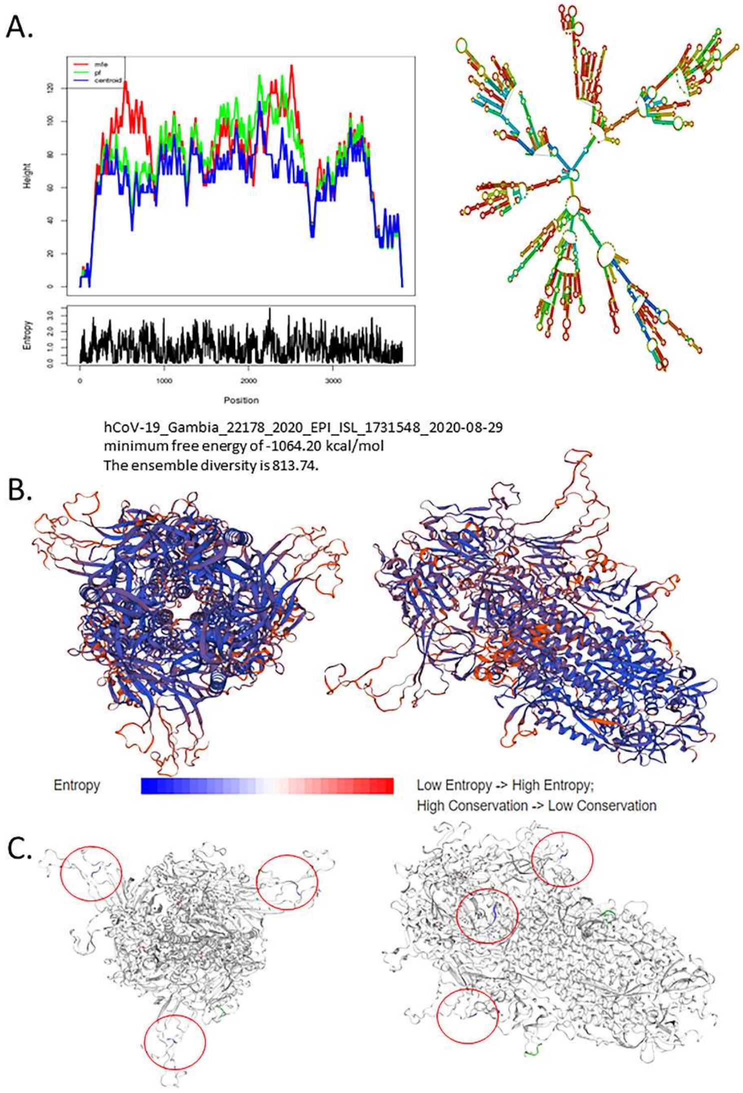

Supplementary Figure 8. A.Secondary structure for the sample hCoV-19_Gambia_22178_2020_EPI_ISL_1731548_2020-08-29, Minimum free energy of -1064.20 kcal/mol, and the ensemble diversity is 813.74. B. 3D structure of the spike protein using the template 7cn8.1.A, seq identity: 92.20% with the description of Glycoprotein Cryo-EM structure of PCoV_GX spike glycoprotein. C. shows multiple deletions in the spike protein marked in blue.

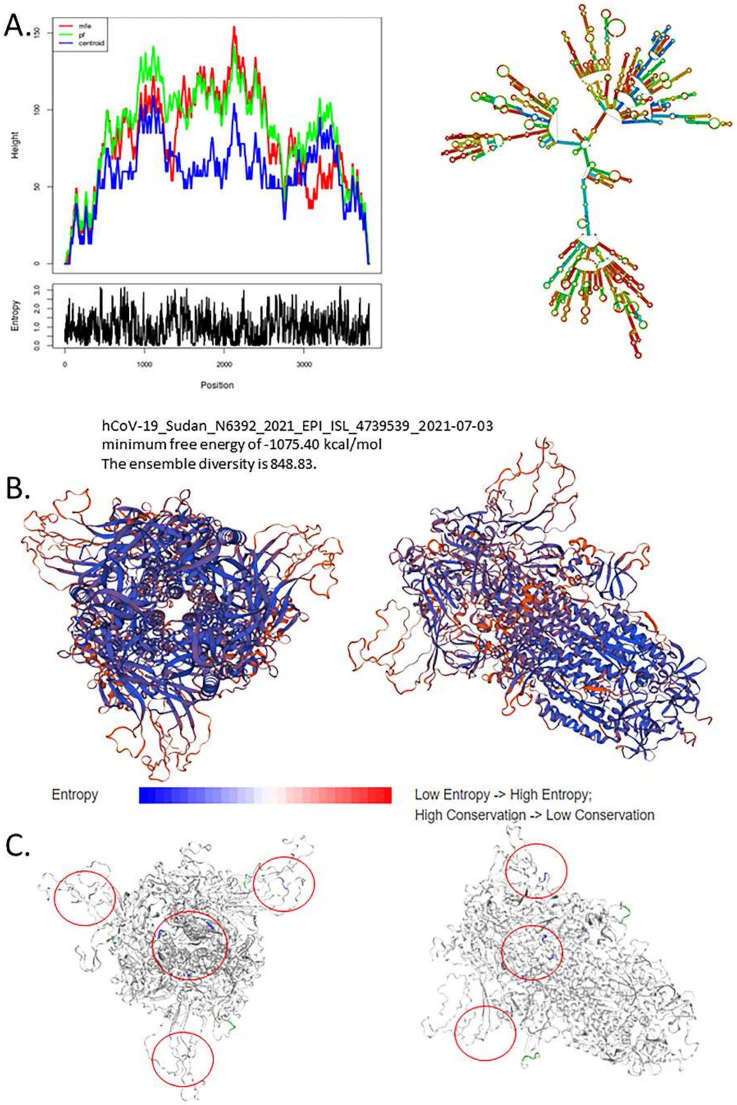

Supplementary Figure 9. A.Secondary structure for the sample hCoV-19_Sudan_N6392_2021_EPI_ISL_4739539_2021-07-03, Minimum free energy of -1075.40 kcal/mol, and the ensemble diversity is 848.83. B. 3D structure of the spike protein using the template 7cn8.1.A, seq identity: 92.08% with the description of Glycoprotein Cryo-EM structure of PCoV_GX spike glycoprotein. C. shows multiple deletions in the spike protein marked in blue.

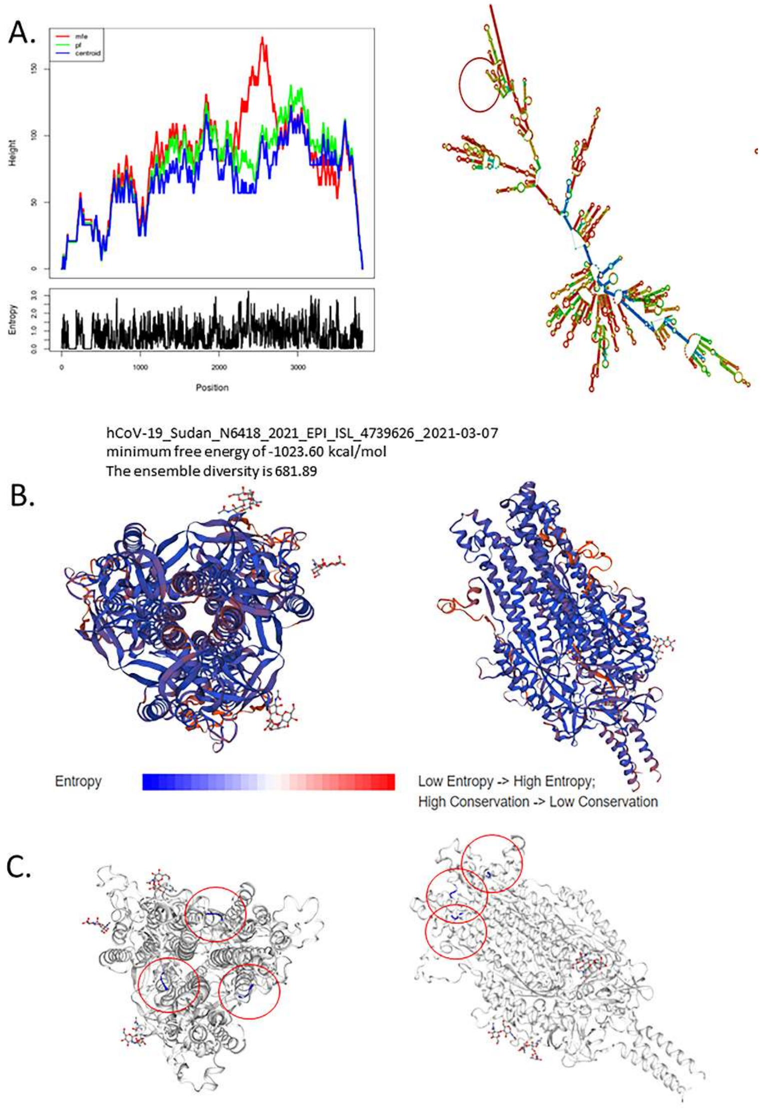

Supplementary Figure 10. A.Secondary structure for the sample hCoV-19_Sudan_N6418_2021_EPI_ISL_4739626_2021-03-07, Minimum free energy of -1023.60 kcal/mol, and the ensemble diversity is 681.89. B. 3D structure of the spike protein using the template 7krs.1.A, seq identity: 100.00% with the description of Spike glycoprotein Structural impact on SARS-CoV-2 spike protein by D614G substitution. C. shows multiple deletions in the spike protein marked in blue.
